# Prognostic significance of systemic immune‐inflammation index in patients with nonfunction pancreatic neuroendocrine tumor undergoing surgical resection

**DOI:** 10.1002/cam4.7114

**Published:** 2024-03-30

**Authors:** Guanhua Chen, Li Liu, Chunlu Tan, Qingquan Tan, Yonghua Chen, Xiangrong An, Xubao Liu, Xing Wang

**Affiliations:** ^1^ Division of Pancreatic Surgery, Department of General Surgery, West China Hospital Sichuan University Chengdu Sichuan China; ^2^ Department of Pediatrics Southwest Medical University Luzhou Sichuan China

**Keywords:** inflammation, nonfunction, pancreatic neuroendocrine tumor, prognosis, systemic immune‐inflammation index

## Abstract

**Purpose:**

The purpose of our study was to investigate the clinical significance and prognostic role of the systemic immune‐inflammation index (SII) in patients who underwent surgical resection for nonfunctioning pancreatic neuroendocrine tumors (pNETs).

**Methods:**

We conducted a retrospective analysis of 364 patients with nonfunctioning pNETs. The association between the SII level and clinical parameters was investigated. The receiver operating characteristic (ROC) curve was used to calculate the optimal SII value. Cox proportional hazard analysis was performed to evaluate the prognostic factors.

**Results:**

Our study included 364 patients with nonfunctioning pNETs who underwent surgery. The median age was 51.0 (43.0, 59.3), and 164 (45.1%) were male. The optimal threshold of SII determined by ROC analysis was 523.95. Higher SII levels were significantly associated with older age (*p* = 0.001), sex (*p* = 0.011), tumor size (*p* = 0.032), and tumor grade (*p* = 0.002). Recurrence was observed in 70 (19.2%) patients following a median follow‐up of 98 months. Univariate analysis showed that higher SII (*p* < 0.0001), tumor size >4 cm (*p* = 0.015), and G2/G3 grade (*p* = 0.002) were significantly associated with disease‐free survival (DFS). Multivariate analysis revealed that higher SII (HR: 7.35; 95% CI: 3.44, 15.70; *p* < 0.0001) and G2/G3 grade (HR: 3.11; 95% CI: 1.42, 6.82; *p* = 0.005) remained significantly associated with tumor recurrence. Furthermore, 46 (12.6%) patients died during the follow‐up. Higher SII (HR: 8.43; 95% CI: 3.19, 22.72; *p* < 0.0001) and G2/G3 grade (HR: 3.16; 95% CI: 1.01, 9.86; *p* = 0.048) were independent predictors of overall survival (OS) by multivariate analysis.

**Conclusion:**

In conclusion, our study revealed that a higher SII level was associated with tumor‐related features (larger tumor size and advanced grade) and subsequent shorter DFS and OS in patients with nonfunctioning pNETs. These results indicated that the SII could serve as an efficient prognostic biomarker for nonfunctioning pNETs.

## 1 INTRODUCTION

Pancreatic neuroendocrine neoplasms (pNENs) are a group of tumors arising from pancreatic neuroendocrine cells and account for only 1%–2% of all pancreatic tumors.^1^ The incidence and prevalence of pNENs are thought to be 0.48 per 100,000 persons and rise annually based on data from surveillance, epidemiology, and end results.^2^ Clinically, PNENs are classified as functional or nonfunctional based on whether they release hormones that cause symptoms. Most pNENs (60%–90%) are nonfunctioning and typically asymptomatic.^3^ The traditionally promulgated perspectives of neuroendocrine neoplasms are indolent tumors. Recent studies suggest that pancreatic neuroendocrine tumors (pNETs) have metastatic potential and are classified as malignant tumors by the World Health Organization (WHO).^4^ The WHO 2017 classification system highlights the association of morphologic differentiation with the definition of well‐differentiated neoplasms (pNETs) and poorly differentiated carcinomas (pancreatic neuroendocrine carcinomas [pNECs]). The grading system for pNETs was classified into three tiers based on proliferation (G1, G2, and G3).^5^, ^6^, ^7^ Furthermore, tumor size,^8^ tumor location,^9^ lymph node invasion,^10^ distant metastasis,^11^ and some related immunologic and histologic biomarkers are believed to be related to prognosis and tumor recurrence. However, these indicators can only be obtained after surgery. Thus, a promising and potential preoperative index is necessary.

The topic of relationships between tumor and inflammation is not new. Related proinflammatory cytokines, such as tumor necrosis factor alpha (TNF‐α), interleukins and interferons released by tumor cells and various inflammatory cells (neutrophils, dendritic cells, macrophages, lymphocytes, etc.), are involved in local and systemic inflammation of tumors.^12^ These inflammatory responses are considered to promote tumor growth and progression, which is associated with tumor prognosis. The systemic immune‐inflammation index (SII) is a reliable, convenient, and stable indicator for evaluating inflammation in the body, as it incorporates three distinct types of inflammatory cells: platelets, neutrophils, and lymphocytes. It is an effective predictor of cancer outcomes in patients with colorectal cancer,^13^ hepatocellular cancer,^14^ gastric cancer^15^ and pancreatic cancer.^16^ However, research regarding the SII in patients with nonfunctioning pNETs is limited. The aim of our study was to investigate the clinical significance and prognostic role of the SII in patients who underwent surgical resection for nonfunctioning pNETs.

## 2 SUBJECTS, MATERIALS AND METHODS

### 2.1 Study design and participants

An analysis of patients undergoing surgical resection for pNETs at West China Hospital from August 2011 to December 2019 was conducted. All patients diagnosed with nonfunctioning pNETs over the age of 18 were included. The exclusion criteria were as follows: (1) synchronous liver metastases, *n* = 11; (2) recent infection, inflammatory diseases, and immune disorder, *n* = 7; (3) functional pNETs, *n* = 32; (4) death within 30 days after surgery, *n* = 3; and (5) incomplete data, *n* = 6. The study was performed in accordance with the Declaration of Helsinki and approved by the Ethics Committee of West China Hospital at Sichuan University.

### 2.2 Data collection

Medical records were abstracted for demographic information, including age, sex, and body mass index. Tumor‐related data, including surgical approach, tumor size and location, Ki‐67 expression level, tumor grade, lymph node invasion status, and resection margin clearance rate, were extracted from the surgical pathology reports. In all patients, we collected neutrophil counts, lymphocyte counts, and platelet counts using an automatic blood cell analyzer (Cysmex, USA) within 1 week before the operation. The SII was calculated as neutrophil × platelet/lymphocyte.

### 2.3 Definitions

Two pathologists independently determined the diagnosis and grade of pNETs, and two other clinicians determined whether the tumor was nonfunctional. Based on ki‐67 recommended by the 2017 WHO classification, the grading system was identified as follows: G1 < 3%, G2 3%–20%, and G3 > 20%.^17^ Nonfunctional pNETs do not present with hormone‐related clinical syndromes, such as hypoglycemia, hyperglycemia with necrolytic migratory erythema, severe watery diarrhea, and Cushing's syndrome.^18^ Surgical approaches include regular resection (pancreaticoduodenectomy and distal pancreatectomy) and irregular resection (enucleation, central pancreatectomy, duodenum‐preserving resection of the pancreatic head, and spleen‐preserving distal pancreatectomy).^19^

### 2.4 Follow‐up

We followed up with patients by reviewing hospital records or contacting their families. Overall survival (OS) refers to the time between surgery and death. From the time of surgery until recurrence, disease‐free survival (DFS) was calculated. Follow‐up was routinely performed every 6 months in the first 2 years after surgery and annually thereafter. Both radiologists and clinicians assessed recurrence based on imaging findings (such as CT, MRI, or PET‐CT) and biopsy results.

### 2.5 Statistical analysis

Categorical variables are expressed as percentages (%), and continuous variables are expressed as medians and interquartile ranges (IQRs). An analysis of the receiver operating characteristic (ROC) curve was performed to determine the area under the ROC curve (AUC), and the Youden Index was used to identify the optimal cutoff values for the SII. The Mann–Whitney *U* test was used to compare continuous variables. The chi‐square test was used to compare categorical variables. An analysis of survival differences was conducted using the log‐rank test based on the Kaplan–Meier method. In this study, a Cox proportional hazard model was used to perform multivariate survival analysis. A *p*‐value < 0.05 was considered statistically significant. All statistical analyses were performed using SPSS 26.0 software.

## 3 RESULTS

### 3.1 Subject characteristics

Table 1 presents the demographic and preoperative characteristics of the 364 patients with nonfunctional pNETs. The median age was 51.0 (43.0, 59.3), and 45.1% (*n* = 164) of them were male. The preoperative median white blood cell level was 6.1 (5.0, 6.9). All patients underwent surgical resection, including 36 (9.9%) with pancreatoduodenectomy, 98 (26.9%) with distal pancreatectomy, 22 (6.1%) with spleen‐preserving distal pancreatectomy, 60 (16.5%) with central pancreatectomy, 58 (15.9%) with enucleation, and 90 (24.7%) with duodenum‐preserving resection of the pancreatic head. Nearly half of the tumors were located in the body and tail (*n* = 198, 54.4%). The median tumor size was 3.0 (1.9, 4.5) cm, and lymph node invasion was present in over 1/3 of patients (*n* = 126, 34.6%). The median ki‐67 was 5.0% (1.0, 7.3). There were 152 patients (41.7%) classified as G1, 160 (44.0%) as G2, and 52 (14.3%) as G3. After a median follow‐up of 98 months (ranging from 38 to 146 months), 46 (12.6%) patients died, and 70 (19.2%) experienced recurrence.

**TABLE 1:** Patients' characteristic.

Variables	
Age (year)	51.0 (43.0, 59.3)
Sex (male, *n*, %)	164 (45.1)
Body mass index (kg/m^2^)	20.7 (19.2, 22.7)
White blood cell (10^11^)	6.1 (5.0, 6.9)
Neutrophil (10^11^)	3.6 (2.9, 4.6)
Lymphocyte (10^11^)	1.6 (1.2, 1.9)
Platelet (10^9^)	189.5 (141.3, 235.3)
Systemic immune‐inflammation index (10^9^)	429.1 (327.5, 523.9)
Surgical process (*n*, %)	
Enucleation	58 (15.9)
Distal pancreatectomy	98 (26.9)
Duodenum‐preserving resection of pancreatic head	90 (24.7)
Central pancreatectomy	60 (16.5)
Spleen‐preserving distal pancreatectomy	22 (6.1)
Pancreatoduodenectomy	36 (9.9)
Tumor size (cm)	3.0 (1.9, 4.5)
T (*n*, %)	
T1	96 (26.4)
T2	144 (39.6)
T3	124 (34.0)
N (*n*, %)	
N0	196 (53.8)
N1	126 (34.6)
Unknown	42 (11.6)
Tumor location (body and tail, *n*, %)	166 (45.6)
Ki‐67 (%)	5.0 (1.0,7.3)
Tumor grade (*n*, %)	
G1	152 (41.7)
G2	160 (44.0)
G3	52 (14.3)
Death (*n*, %)	46 (12.6)
Recurrence (*n*, %)	70 (19.2)

### 3.2 ROC analysis

**FIGURE 1:**
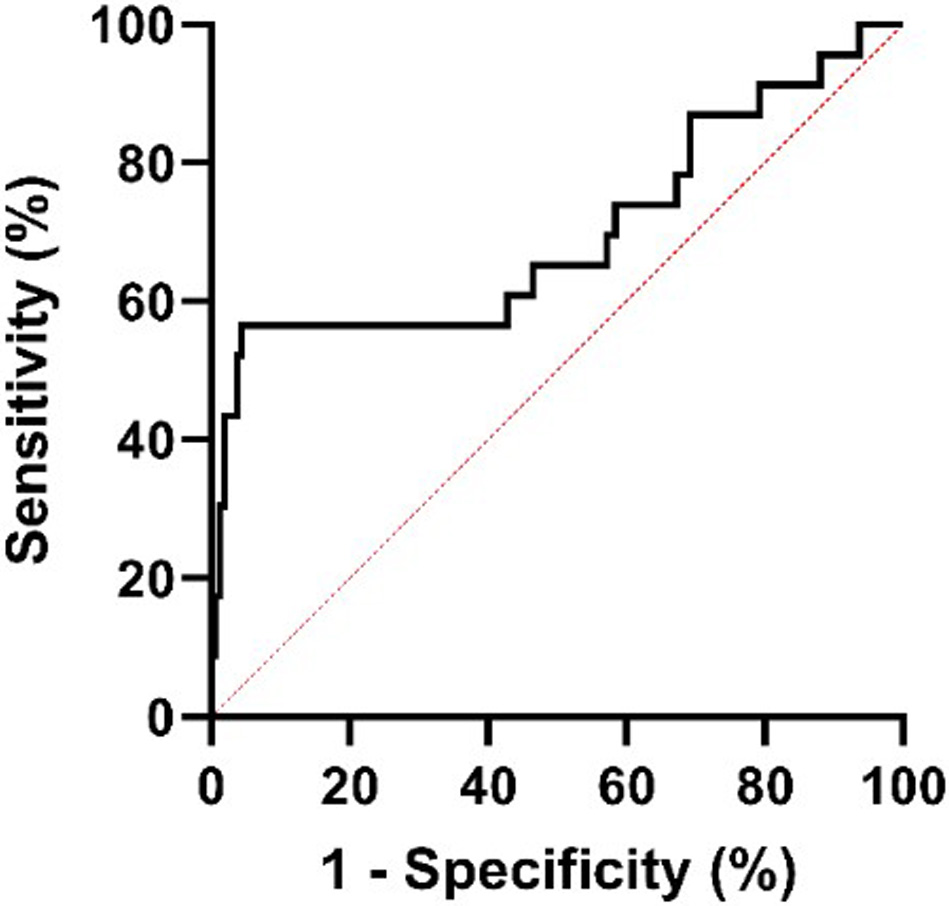
Receiver operating curve analysis of disease‐free survival for systemic immune‐inflammation index (SII) (AUC: 0.70, 95% CI: 0.56, 0.84).

Using tumor recurrence as the end point, as shown in Figure 1, ROC analysis was performed to identify the optimal cutoff point, which was 523.95 for the SII (AUC: 0.70, 95% CI: 0.56 0.84). According to the SII level, patients were divided into two groups (high SII group: SII > 523.95 and low SII group: SII < 523.95) for further analysis.

### 3.3 SII level and clinical characteristics

The clinical parameters stratified by the SII level are shown in Table 2. Taking the SII (523.95) as a cutoff point, we divided the patients into the low SII group and the high SII group. Higher SII levels were significantly associated with older age (*p* = 0.001) and sex (*p* = 0.011). Patients with tumor sizes <2, 2–4, and >4 cm accounted for 29.4% (*n* = 80), 39.7% (*n* = 108), and 30.9% (*n* = 84) in the low SII group and 16.7% (*n* = 16), 39.1% (*n* = 36), and 43.5% (*n* = 40) in the high SII group, respectively. There was a notable disparity in the dimensions of tumor size (*p* = 0.031). Furthermore, a significant difference in tumor grade (*p* = 0.002) was observed in the high SII group. In contrast, the SII level was not associated with lymph node invasion, BMI, surgical approach, tumor location, or resection margin.

**TABLE 2:** The correlation between the systemic immune‐inflammation index and clinical parameters.

Variables	Low SII group	High SII group	*p*
Age			
<51	150 (55.1)	32 (34.8)	0.001
>51	122 (44.9)	60 (65.2)	
Sex			
Female	160 (58.8)	40 (43.5)	0.011
Male	112 (41.2)	52 (56.5)	
Body mass index (kg/m^2^)	20.61 (18.79, 22.63)	21.27 (19.44, 23.08)	0.243
Tumor location			
Head and neck	152 (55.9)	46 (50)	0.327
Body and tail	120 (44.1)	46 (50)	
Tumor size			
<2 cm	80 (29.4)	16 (16.7)	0.031
2–4 cm	108 (39.7)	36 (39.1)	
>4 cm	84 (30.9)	40 (43.5)	
Lymph node invasion			
No	152 (55.9)	44 (47.8)	0.291
Yes	88 (32.4)	38 (41.3)	
Unknown	32 (11.8)	10 (10.9)	
Tumor grade			
G1	126 (46.3)	26 (28.3)	0.002
G2/G3	146 (53.7)	66 (71.7)	
Resection margin			
R1	6 (100)	0 (0)	0.151
Surgical process			
Regular resection	61	73	0.441
Irregular resection	118	112	

### 3.4 DFS and the SII

**FIGURE 2:**
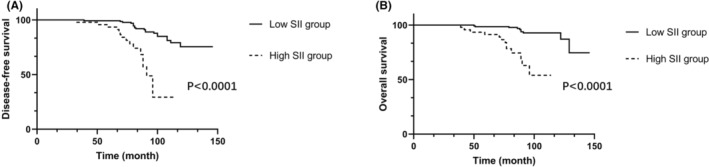
Kaplan–Meier curve of disease‐free survival (A) and overall survival (B) stratified by systemic immune‐inflammation index level.

In addition, we evaluated the utility of the preoperative SII to identify patients with postoperative recurrence (Figure 2A). The 5‐year DFS rates were 98.5% and 93.4%, with 10‐year DFS rates of 75.5% and 29.3%, respectively, in the low and high SII groups. As shown in Table 3, univariate analysis identified higher SII >523.95 (HR: 6.23; 95% CI: 3.06, 12.69; *p* < 0.0001), tumor size >4 cm (HR: 3.04; 95% CI: 1.24, 7.48; *p* = 0.015), and G2/G3 grade (HR: 3.33; 95% CI: 1.57, 7.10; *p* = 0.002) as significantly associated with DFS. After multivariate analysis, higher SII (HR: 8.43; 95% CI: 3.19, 22.72; *p* < 0.0001) and G2/G3 grade (HR: 3.11; 95% CI: 1.42, 6.82; *p* = 0.005) remained significantly associated with tumor recurrence.

**TABLE 3:** Univariate and multivariate Cox regression analysis of disease‐free survival.

	Univariate analysis		Multivariate analysis	
	HR (95% CI)	*p*	HR (95% CI)	*p*
Age (>51)	1.11 (0.58, 2.13)	0.759		
Gender (male)	1.13 (0.59, 2.18)	0.713		
BMI	1.02 (0.89, 1.17)	0.759		
SII (>523.95)	6.23 (3.06, 12.69)	<0.0001	7.35 (3.44, 15.70)	<0.0001
Surgical process (irregular resection)	1.05 (0.53, 2.08)	0.881		
Tumor size				
<2 cm	Ref			
2–4 cm	1.49 (0.59, 3.73)	0.397	1.22 (0.48, 3.08)	0.682
>4 cm	3.04 (1.24, 7.48)	0.015	2.33 (0.91, 5.95)	0.072
Lymph node invasion (positive)	1.74 (0.89, 3.38)	0.104		
Tumor grade (G2/G3)	3.33 (1.57, 7.10)	0.002	3.11 (1.42, 6.82)	0.005
Tumor location (body and tail)	0.77 (0.40, 1.50)	0.447		

Abbreviations: CI, confidence intervals; HR, hazard ratio; Ref, reference.

### 3.5 OS and SII

Figure 2B shows the Kaplan–Meier curves of OS of the patients with low SII levels and high SII levels. After a median follow‐up of 98 months (ranging from 38 to 146 months), the 5‐year OS rates were 99.3% and 95.6%, while the corresponding 10‐year OS rates were 92.8% and 53.4% in the low and high SII groups, respectively (*p* < 0.0001). We used the Cox proportional hazard model to analyze the clinical variables in relation to OS (Table 4). According to univariate analysis, age > 51 (HR: 2.53; 95% CI: 1.04, 6.16; *p* = 0.041), higher SII >523.95 (HR: 8.25; 95% CI: 3.35, 20.35; *p* < 0.0001), tumor size >4 cm (HR: 3.05; 95% CI: 1.04, 8.93; *p* = 0.043), lymph node invasion (HR: 2.45; 95% CI: 1.06, 5.67; *p* = 0.037), and tumor G2/G3 grade (HR: 3.68; 95% CI: 1.30, 10.51; *p* = 0.015) significantly decreased OS. Multivariate analysis revealed that higher SII > 523.95 (HR: 8.43; 95% CI: 3.19, 22.72; *p* < 0.0001) and G2/G3 grade (HR: 3.16; 95% CI: 1.01, 9.86; *p* = 0.048) were independent predictors of OS.

**TABLE 4:** Univariate and multivariate Cox regression analysis of overall survival.

	Univariate analysis		Multivariate analysis	
	HR (95% CI)	*p*	HR (95% CI)	*p*
Age (>51)	2.53 (1.04, 6.16)	0.041	1.56 (0.61, 3.97)	0.350
Gender (male)	1.58 (0.70, 3.59)	0.273		
BMI	1.13 (0.97, 1.33)	0.122		
SII (>523.95)	8.25 (3.35, 20.30)	<0.0001	8.43 (3.19, 22.72)	<0.0001
Surgical process (irregular resection)	0.53 (0.20, 1.42)	0.205		
Tumor size				
<2 cm	Ref		Ref	
2–4 cm	0.90 (0.27, 2.94)	0.855	0.71 (0.21, 2.42)	0.584
>4 cm	3.05 (1.04, 8.93)	0.043	2.20 (0.67, 7.16)	0.192
Lymph node invasion (positive)	2.45 (1.06, 5.67)	0.037	1.47 (0.60, 3.62)	0.399
Tumor grade (G2/G3)	3.68 (1.30, 10.51)	0.015	3.16 (1.01, 9.86)	0.048
Tumor location (body and tail)	0.62 (0.25, 1.51)	0.290		

Abbreviations: CI, confidence intervals; HR, hazard ratio; Ref, reference.

## 4 DISCUSSION

The primary treatment for pNENs is surgery^20^; however, the postoperative prognosis varies among different grades and clinical stages. In addition, various clinically related factors have been studied for their prognostic value in pNENs, including tumor size, location, tumor grade, perineural invasion, lymph node metastasis, and vascular involvement.^21^ However, most of these factors are acquired postoperatively, which limits their preoperative utility. A convenient, economical, and reliable preoperative index remains elusive. The bidirectional relationship between inflammation and tumors, particularly those originating from the gastrointestinal tract, has been extensively documented in recent years.^22^, ^23^, ^24^ The inflammatory response and related cytokines not only facilitate tumorigenesis but also promote tumor progression.^24^ Previous studies have demonstrated a higher incidence of neuroendocrine tumors in chronic inflammation,^25^, ^26^ implying their potential correlation. The SII is considered to be a reliable biomarker in relation to local and systemic inflammatory responses.^27^ In our study, the higher SII level was significantly associated with age (*p* = 0.001), sex (*p* = 0.011), tumor size (*p* = 0.031), and tumor grade (*p* = 0.002). Furthermore, an elevated SII level was found to be inversely correlated with OS (*p* = 0.014) and DFS (*p* = 0.027), indicating its potential as an efficient predictor for tumor prognosis and recurrence.

The SII biomarker, a novel inflammatory biomarker, not only better reflects host immunity in cancer patients but is also easily accessible.^28^ It comprises three crucial types of inflammatory cells, including neutrophils, lymphocytes, and platelets. Neutrophils are crucial inflammatory cells in the body that can be recruited by tumor cells during early stages and initiate tumorigenesis.^29^ Rich granules in neutrophils, such as MMP‐9 and ARG‐1, have been shown to promote tumor growth.^30^ Furthermore, the activation of neutrophils in certain types of cancer may reactivate dormant cancer cells, ultimately leading to tumor recurrence.^31^ From the cohort study conducted by Wu‐Hu Zhang, it was found that, in pNETs, patients with a higher count of tumor‐infiltrating neutrophils exhibited more lymph node invasion, perineural invasion, and advanced stage, and higher tumor‐infiltrating neutrophils were linked to reduced DFS and OS.^32^

In general, lymphocytosis is associated with a better prognosis since lymphocytes play a crucial role in the host's anticancer immune response.^33^ Some studies have also shown that tumor‐infiltrating lymphocytes exhibit antitumor immunity.^34^ A study from YiTao Gong demonstrated that, in pNETs patients with distant metastasis tended to have lower total T cells in the peripheral blood and that a low B‐cell count may predict poor DFS.^35^

The role of platelets in tumor progression comes into sight. Increased circulation of activated platelets occurs as a result of the release of bioactive substances such as interleukin‐6 and tissue factor. On the other hand, an activated thrombocyte sets up granules such as vascular endothelial growth factor, platelet‐derived growth factor and transforming growth factor‐β free, thereby promoting tumor growth.^36^ The study conducted by Xu SS indicated that, in both OS and DFS, platelets were an independent prognostic factor in resectable pNETs.^37^

It has been commonly known that inflammation can promote tumor progression and recurrence, thus, has an adverse impact on survival. Recently, several studies have demonstrated that inflammatory markers could serve as reliable predictors for tumor prognosis by reflecting the level of the inflammatory response. The neutrophil‐to‐lymphocyte ratio (NLR) and platelet‐to‐lymphocyte ratio (PLR) have been extensively investigated and shown to be associated with OS and DFS in various types of cancer, including gastric cancer,^38^ hepatocellular carcinoma,^39^ and pancreatic cancer.^40^, ^41^ Compared to NLR and PLR, SII is an accurate systemic inflammatory index based on neutrophil, lymphocyte, and platelet counts, which is more comprehensive in reflecting inflammation status. Recently, the SII has been reported to show superior predictive value for the prognosis of patients with pancreatic ductal adenocarcinoma compared with PLR and NLR.^16^, ^42^ Moreover, a higher SII is an independent predictive indicator of postoperative liver metastasis for patients with colorectal cancer.^43^ From a study by Dr. Wang, they found that a higher level of SII is an adverse prognostic factor for hepatocellular carcinoma patients who undergo hepatectomy.^44^ In addition, a higher SII is an adverse prognostic factor for patients with gastroesophageal adenocarcinoma.^45^ Overall, the relationship between the SII and tumor prognosis may result from thrombocythemia, neutrophilia, and lymphopenia, which suggests an elevated inflammatory status and decreased immune system response.

In our study, we aimed to minimize heterogeneity by selecting subjects with nonfunctional pNETs and no distant metastasis. Our findings indicate that a higher SII is associated with reduced DFS and OS, suggesting its potential utility as a prognostic indicator in nonfunctioning pNETs. The impact of the SII on OS may be attributed to the intrinsic characteristics of the tumor. According to our data, a higher SII level was associated with age, sex, a larger tumor size, and advanced grade. However, our findings did not reveal any impact of age, sex or tumor size on DFS and OS, which may be attributed to the heterogeneity or limited sample size in our study.

The study has certain limitations, including its retrospective nature and single‐center design. Therefore, it is recommended that multicenter studies should be conducted to provide more robust evidence. Additionally, while the prognostic value of the SII was confirmed, further investigation comparing the SII with other inflammatory biomarkers, such as the NLR and PLR, would be beneficial. Last, the sample size was limited to nonfunctional pNETs without distant metastasis; larger samples encompassing different stages are necessary for a comprehensive understanding of this topic.

## 5 CONCLUSIONS

In conclusion, our study revealed that a higher SII level was associated with tumor‐related features (larger tumor size and advanced grade) and subsequent shorter DFS and OS in patients with nonfunctioning pNETs. These results indicated that the SII could serve as an efficient prognostic biomarker for nonfunctioning pNETs.

## AUTHOR CONTRIBUTIONS

**Guanhua Chen:** Conceptualization (equal); data curation (equal); formal analysis (equal); investigation (equal); methodology (equal); resources (equal); software (equal); writing – original draft (equal); writing – review and editing (equal). **Li Liu:** Formal analysis (equal); investigation (equal); methodology (equal). **Chunlu Tan:** Data curation (equal); investigation (equal). **Qingquan Tan:** Conceptualization (supporting); formal analysis (supporting); investigation (equal). **Yonghua Chen:** Formal analysis (equal); investigation (equal); methodology (supporting). **Xiangrong An:** Conceptualization (equal); investigation (equal); methodology (supporting); software (equal). **Xubao Liu:** Data curation (equal); formal analysis (equal); investigation (equal); writing – review and editing (equal). **Xing Wang:** Conceptualization (lead); formal analysis (equal); investigation (equal); methodology (equal); supervision (lead); validation (lead); writing – original draft (equal).

## CONFLICT OF INTEREST STATEMENT

The authors have no relevant financial or nonfinancial interests to disclose, and informed consent was obtained from all individual participants included in the study.

## ETHICS STATEMENT

This study was performed in line with the principles of the Declaration of Helsinki. Approval was granted by the Ethics Committee of West China Hospital, Sichuan University (Date: August 2019; Number: 2019‐735).

## Data Availability

The datasets generated during and analyzed during the current study are available from the corresponding author on reasonable request.
